# The introduction of tobacco excise taxation in the Gulf Cooperation Council Countries: a step in the right direction of advancing public health

**DOI:** 10.1186/s12889-022-13190-0

**Published:** 2022-04-13

**Authors:** Sofia Delipalla, Konstantina Koronaiou, Jawad A. Al-Lawati, Mohamed Sayed, Ali Alwadey, Ejlal F. AlAlawi, Kholoud Almutawaa, Amal H. J. Hussain, Wedad Al-Maidoor, Yahya M. Al-Farsi

**Affiliations:** 1 University of Macedonia, Thessaloniki, Greece; 2 Ministry of Health, Muscat, Sultanate of Oman; 3 Gulf Health Council, Riyadh, Kingdom of Saudi Arabia; 4 Ministry of Health, Riyadh, Kingdom of Saudi Arabia; 5 Ministry of Health, Manama, Kingdom of Bahrain; 6 Ministry of Public Health, Doha, State of Qatar; 7 Ministry of Health, Kuwait City, State of Kuwait; 8 Ministry of Health, Abu Dhabi, United Arab Emirates; 9 Sultan Qaboos University, Muscat, Sultanate of Oman

**Keywords:** Cigarette tax, excise tax reform, GCC countries, Saudi Arabia, public health policy

## Abstract

**Background:**

The Gulf Cooperation Council (GCC) countries relied, until recently, solely on import duties for tobacco products. The agreement for the introduction of an excise and value added tax (VAT) in 2016 and 2017, respectively, in most GCC countries, was a major breakthrough for public health. There is, however, ample room for improvement.

**Methods:**

The study examines the outcomes of tax reforms, for both public health and public finances, based on the World Health Organization (WHO) recommendations and best practices worldwide. Tax simulations were performed using the WHO TaXSiM model. The study is based on data from Saudi Arabia, the only GCC country for which sufficient data existed.

**Results:**

We recommend a stepwise tax reform, which involves increasing the current ad valorem excise tax rate, phasing out import duties keeping total tax share constant and introducing a minimum excise, and finally switching to a revenue-neutral specific excise. Specific excises must be adjusted for inflation and income increases. If implemented, cigarette tax reform simulations show that the recommended reforms would lead to a higher than 50% increase in cigarette prices, 16% reduction in cigarette sales and almost 50% increase in total cigarette tax revenue. A significant number of cigarette-related deaths would be averted.

**Conclusions:**

The recommended tax reforms are expected to lead to significant improvements in both public health and tobacco tax revenues. Our results provide useful insights that are of relevance to the whole GGC region. The effectiveness of the reforms, however, requires a strong tax and customs administration, including the establishment of a good database to monitor and advance public health.

## Background

Design and implementation of tobacco taxation is the most efficient and cost-effective measure to control tobacco consumption [1, 2]. When significant tax increases are designed and implemented based on the general directions and best practices presented in the WHO Technical Manual on Tobacco Tax Policy and Administration [3], they lead to price increases which have a beneficial effect on consumers' behaviour. Taxes, as part of a comprehensive tobacco control strategy, bring about price increases which reduce tobacco use and the associated negative health effects it causes. Tobacco use is a major risk factor for many noncommunicable diseases (NCDs), which lead to a reduction in personal levels of well-being as well as an increase in economic costs. In the Gulf Cooperation Council (GCC) countries, total cost of smoking and second-hand smoke is estimated to amount to 1.04% of total Gross Domestic Product (GDP) in 2017 [4]. The highest percentage of direct cost (health expenditure) is government health spending, and the highest proportion of indirect cost (productivity losses due to morbidity and mortality) results from smoking by men and middle-aged people. The main causes are cardiovascular diseases, for mortality cost, and type 2 diabetes mellitus, for morbidity cost. Excise taxes help improve public health and reduce tobacco-related health expenditure whilst, simultaneously, generating considerable tax revenue [3].

Before the introduction of excises, the GCC countries relied solely on import duties, putting both revenues and public health at risk due to the pressure of an increasing number of free trade agreements. An import duty of a 100% of the cost, insurance and freight (CIF) value applied on all tobacco products, together with a minimum duty amount (whichever is highest). In 2016, the minimum import duty was doubled. Kuwait was, and still remains, the only GCC country which did not double the minimum import duty on tobacco products [5].

In 2016, the GCC countries collectively agreed to implement a harmonized excise tax at the rate of 100% of (excise-exclusive) retail price on all tobacco products [6]. The excise was first introduced in Saudi Arabia in June 2017, followed by the UAE and Bahrain in October and December 2017, respectively. In January 2019, the tax was implemented in Qatar, and 5 months later in Oman. Implementation of excise in Kuwait was deferred to the 2020–21 fiscal year. In 2017, GCC countries have also agreed on imposing a value added tax (VAT) on all goods and services [7]. Saudi Arabia and the UAE implemented VAT in January 2018 and Bahrain in January 2019. There are ongoing preparations for VAT implementation in Qatar and Oman in 2021 [8, 9], while Kuwait has not as yet set a date for VAT implementation.

As a consequence of the tax reform, retail volume sales of cigarettes at the GCC level, whilst steadily increasing until 2016, decreased sharply in 2017, according to Euromonitor [10]. This is mainly due to a decrease in retail volume in Saudi Arabia, as it represents 64% of the GCC retail volume and was the first country to introduce tobacco excises. The UAE market also contributed to this reduction but to a lesser degree [10]. Oxford Economics estimated that cigarette tax revenue across Kuwait, Oman, Saudi Arabia and United Arab Emirates increased by 66.7% in 2017 relative to the previous year [11]. This increase seems to be entirely due to the introduction of excise taxation, since legal sales decreased.

In all GCC countries, cigarettes became less affordable since 2008, with an increase in the Relative Income Price (RIP) (a measure of affordability) in the range of 9.65% in Kuwait to 15.38% in Saudi Arabia (Table 1). The price dispersion index ranges from 20.45% in Oman to 54.76% in the UAE (Table 1). This means that the price of the most expensive brand is 1.83 (UAE) to 4.89 (Oman) times higher than the price of the cheapest brand. As the index increases, the gap between cheapest and most expensive brands decreases and, thus, the opportunities to switch to cheaper brands are fewer [12].

**Table 1:** Relative Income Price (RIP) and Price Dispersion Index, 2020

		Bahrain	Kuwait	Oman	Qatar	SA	UAE
**Affordability**	Affordability Index	2.67%	1.27%	3.97%	1.15%	3.81%	1.79%
	Trend growth rate in affordability 2008–2018	11.54%	9.65%	13.61%	14.24%	15.38%	13.06%
	Cigarettes less affordable since 2008	YES	YES	YES	YES	YES	YES
**Price Dispersion**	Price of cheapest brand, pack of 20, in local currency and (USD)	0.80 (2.13)	0.30 (0.98)	0.45 (1.17)	9.00 (2.47)	14.00 (4.00)	11.50 (2.31)
	Price of Marlboro (or other premium brand), pack of 20, in local currency and (USD)	2.30 (6.12)	0.85 (2.78)	2.20 (5.72)	22.00 (6.04)	28.00 (7.47)	21.00 (5.72)
	Price dispersion index	34.78%	35.29%	20.45%	40.91%	50.00%	54.76%

Source: WHO Report on the Global Tobacco Epidemic,2021, Web Annex Appendix VI (12). Corrections have been made regarding the price of the cheapest brand in SA and the UAE, after communication with WHO EMRO. SA, Saudi Arabia; UAE, United Arab Emirates; Prices in Bahrain, Saudi Arabia and UAE include excise taxes

RIP is defined as the percentage of per capita GDP required to buy 2000 cigarettes of the most sold brand in a specific year (12). Price dispersion index is defined as the price of the cheapest brand as a percentage of the price of the most expensive one

In 2018, the sum of excise and import duty as a percentage of final price (all taxes inclusive) of the most sold brand was lower than 75%, which is recommended by the World Health Organization (WHO) [3, 13]. Specifically, in Bahrain, Saudi Arabia and the UAE, which implemented an excise tax in 2018, the share of total tax in final price was 70, 68.09 and 73.54%, respectively. For the remaining countries, where only import duties were implemented, the duty share was extremely low: 21.2% in Kuwait, 25% in Oman and 40% in Qatar. Low tax shares have hardly any effect on consumption and do not exploit the full potential for revenue raising.

The GCC agreement for the introduction of an excise tax and VAT in 2016 and 2017, respectively, was a step in the right direction. Opportunities for improvement, however, still exist. Our aim here is to examine a three-step cigarette tax reform, based on the WHO recommendations as well as best practices followed by countries that adopted successful tobacco tax policies [13, 14], and estimate its impact on consumption, prevalence, tobacco-related deaths and tax revenue.

### Tobacco tax structure and rates in Saudi Arabia

In 2016, when only import duty applied (100% of CIF value), the estimated (sales-weighted) average tax-inclusive retail sales price (TIRSP) of a pack of 20 was Saudi Rials (SAR) 12.60 (Table 2). Import duty constituted 40% of the average TIRSP. The amount of import duty remained the same in all examined years as the calculation base is the CIF value, and this was assumed unchanged.

**Table 2:** Estimated cigarette market indicators (averages), in local currency, 2016–2018, Saudi Arabia

Cigarette market indicators (Averages)	2016	2017	2018
**Final price**	**12.6**	**25.6**	**26.0**
**Import duty (SAR)**	5.0	5.0	5.0
**Excise tax (SAR)**	0.0	12.8	12.4
**VAT (SAR)**	0.0	0.0	1.2
**Import duty as % of final price**	39.7	19.5	19.2
**Excise tax as % of final price**	0.0	50.0	47.7
**VAT % of final price**	0.0	0.0	4.6
**Total tax excl. VAT as % of final price**	39.7	69.5	66.9
**All tax as % of final price**	39.7	69.5	71.5

Estimations based on data from Euromonitor International (2018) and government sources. VAT: value added tax; SAR: Saudi Arabia currency in Rials (1 SAR ~ US $0.27)

In 2017, excise was introduced and the estimated average TIRSP increased to SAR 25.60. The excise tax was 50% of the average TIRSP and share of import duty decreased to 20%. Thus, total tax increased to 70% of the average TIRSP. Finally, in 2018, VAT was implemented, increasing the estimated average TIRSP to SAR 26 and total tax (including VAT) to 71.5% of the average TIRSP.

There was an upward trend on both total and legal sales until 2016 [10]. In 2017, both total and legal sales decreased by 19.5 and 21%, respectively. The difference between total and legal sales is the illegal as well as free trade zone (FTZ) sales, and they were estimated to be 5 to 7% of total sales [10]. Oxford Economics, compared to Euromonitor, underestimates the sum of illicit and FTZ sales for the years before the introduction of the excise. In the second quarter of 2018, however, they report a rapid increase of illicit and FTZ sales, reaching 10.7% of total sales. Regarding cigarette data, let us keep in mind that the main Euromonitor source is the tobacco industry itself and that the Oxford Economics report was funded by the tobacco industry [11].

## Methods

We considered a three-year reform, starting with a straightforward scenario for immediate action, and then continued with a mid-term scenario that possibly involves lengthy procedures such as amendments in the GCC Treaties [6, 7]. Keeping in mind that all tobacco products are harmful and should be taxed comparably, we focus on reforms on the rate and structure of the cigarette excise, since data did not exist for other tobacco products. Not much is lost, however, as cigarettes are the most common tobacco product used in GCC countries, although waterpipe tobacco is also used [15, 16].

We analyze the following tax reforms for immediate to medium term action. In the first year, increase the tax rate (excise plus import duty) to be at least 75% of final retail price (all taxes-inclusive). In the second year, gradually replace import duties, increasing the excise tax rate to compensate, and introduce a minimum excise tax (MET). As the global trend is to reduce trade barriers, it is best to replace import duties with domestic taxes to compensate for revenues lost. In this case, as minimum import duties are not in place anymore, a MET per 1000 cigarettes or pack of 20 should be introduced. The MET guarantees a significant increase in price, especially in the lowest price segment, and hence in health benefits. In the third year, the reform would be completed by a gradual switch to specific excise keeping tax revenue constant. An ad valorem component, of course, will still apply through VAT.

According to global evidence [13], the less preferable tax type, from a public health perspective, is the ad valorem tax. It is not only more likely to lead to lower average prices but also, by increasing the gap between lower- and higher-priced brands, encourages substitution towards cheaper brands. In terms of administration, since we need to know both the volume and value of taxed products, ad valorem taxation provides incentives for product undervaluation to reduce tax liability. As a result, tax revenue is less stable and more difficult to forecast. Given these issues, the WHO recommends a specific excise tax or a mixed excise with a minimum excise tax (MET). If the real value of specific excises is likely to erode over time, countries must adjust it for price inflation or income increases. Regarding the base for the ad valorem component, retail price is preferable than producer (or import) price, since it is easier to observe and less likely to be manipulated. Finally, for significant price increases, the sum of excise and import duty as a percentage of final price (all taxes inclusive) of the most sold brand is recommended to be higher than 75% [3]. Countries that follow these best practices have the highest prices and hence the highest beneficial impact on consumer behaviour [13].

To test these tax reforms, we performed simulations for Saudi Arabia. All tobacco products are imported since tobacco cultivation and production is banned locally [17]. Cigarette market is characterized by the dominance of premium brands and Marlboro is the most popular brand of the category as well as of the market as a whole [10].

To estimate the outcomes of the suggested tax policy reforms on cigarette market and tax revenues, we used the WHO TaXSiM model, which requires detailed data on sales, retail and producer price and all types of taxes (import duties, excises and VAT) per brand, as well as country population and adult smoking prevalence [18]. The model predicts the impact of changes in the tax structure and/or tax rates on the retail price, consumption, excise and total tax revenues generated by each brand and market segment, as well as smoking prevalence. The more detailed information available, the more accurate the predictions. In most cases, information regarding consumer and producer response to tax increases is not available, and certain assumptions have to be made.

The GCC countries only recently started to collect price data by cigarette brands. Sales by brand, however, are more difficult to find. Hence, prices and sales for most of the cigarette brands (covering just above 90% of the market) were provided by Euromonitor [10]. Tax information was provided by government. Data on population are available from Saudi Arabia’s governmental statistical office [19]. In 2018, cigarette smoking prevalence was reported to be 32.5% in males and 3.9% in females, based on a latest study [20].

There are no studies estimating behaviour of either demand or supply side in the GCC tobacco market. Thus, we used demand elasticity values consistent with the global evidence that, in high income countries, the price elasticity of demand is on average − 0.4, ranging from − 0.2 to − 0.6 [1, 2]. In the Saudi Arabia cigarette market, the market share of premium, medium-priced and economy brands was 62, 21 and 17%, respectively, in 2017. The market is dominated by the premium brand so we assumed a less elastic demand than the global average (− 0.3). We assume demand for premium (economy) brands is less (more) sensitive to price changes. We made conservative assumptions regarding cigarette demand elasticity for three price segments, to estimate a lower bound in consumption change. However, we also performed a sensitivity analysis assuming higher elasticities per price segment. Distribution margins and CIF are assumed to have remained constant; any changes in final retail price are entirely due to changes in tax structure and/or tax rate. The tax is assumed to be fully passed on to consumer prices. Given that data on demand behaviour are not available, it is also initially assumed that consumers do not trade up or down (that is, switch to more or less expensive brands) in response to price increases.

## Results

Starting with the first part of the reform for immediate application (year 1), we increased the excise tax rate such that total tax (excluding VAT) is equal to 75% of TIRSP. As excise rate increases, import duty rate is gradually phased out. The average excise per pack increased by 88% and that resulted in 44% increase in average price (Table 3).

**Table 3:** Simulated tax effects on consumption, revenue and number of smokers in Saudi Arabia

	***Model predictions***		
	***Year 1***	***Year 2***	***Year 3***
**Average cigarette pack price (SAR)**	38	40	41
**Average total tax per pack**	30	33	33
**Average excise per pack**	23	31	31
**Change in price per pack**	44%	8%	1%
**Change in average excise per pack**	88%	33%	1%
**Import duty as % of final price**	13%	–	–
**Excise tax as % of final price**	62%	76%	76%
**Total tax as % of final price**	80%	81%	81%
***Assume: e (premium) = −0.2; e (mid-price) = − 0.3; e (economy) = − 0.4***			
**Change in number of smokers**	- 5%	−2%	-0.3%
**Change in prevalence**	- 1%	-0.4%	-0.1%
**Change in sales**	-11%	−4%	−1%
**Change in excise revenue**	67%	27%	0%
**Change in VAT revenue**	28%	3%	5%
**Change in import duty revenue**	-11%	*–*	–
**Change in total tax revenue**	44%	4%	0.2%
**Change in industry revenue**	-10%	−2%	−0.3%

Simulations are performed using the tax simulation model developed by the WHO (WHO TaxSim), with 2018 as the baseline year. Estimations are based on Euromonitor data for Saudi Arabia for December 2017 and government sources. *VAT* Value added tax, *SAR* Saudi Arabia currency in Rials (1 SAR ~ US $0.27); e: own price elasticity of demand for premium, mid-price and economy brands

Cigarette sales and industry revenue are expected to fall by 11 and 10%, respectively. The change in tax revenue is expected to be even more pronounced. Specifically, excise and VAT revenue are expected to increase by 67 and 28%, respectively. Import duty revenue, however, as expected, will be reduced by 11%. The total tax revenue will increase by 44%. The number of cigarette smokers is expected to decline by 5% and the overall smoking prevalence will fall by 1%.

Due to the ad valorem nature of the excise tax, changes in key market indicators are expected to be more pronounced for premium brands and smaller for economy brands. The price dispersion index is 38%, since the most expensive brand is estimated at SAR 47.37 and the cheapest brand at SAR 18.05. The price dispersion index is relatively low, creating opportunities for trading down.

On average, excise revenue increases by 67%, but the corresponding increase per price segment is 70% for premium, 62% for mid-priced and 54% for economy brands. Post tax reform, total tax is around 80% of TIRSP on average, with this share being higher for low-priced cigarettes (around 87%) due to the minimum import duty to which they are subjected.

The next step (year 2, in Table 3) involves replacing import duties with excise duty keeping total tax share (excluding VAT) constant, that is, set excise tax at 75% of TIRSP and introduce a MET at 70% of weighted average price (WAP). The introduction of MET (SAR 28) has an impact on both mid-priced and economy brands. Excise tax as percentage of TIRSP is 76% for mid-priced and 87% for economy brands. Thus, MET has a significant effect especially on economy brands.

This reform would lead to a further 8% increase in average price, 4% reduction in sales, 4% increase in total cigarette tax revenue and, more specifically, 27% increase in excise revenue and 3% increase in VAT revenue. Furthermore, it will lead to 2% reduction in number of smokers with 0.4% reduction in smoking prevalence.

Finally (year 3, in Table 3), a switch to a revenue-neutral specific tax rate is examined. The ad valorem rate is replaced by a specific excise such that excise tax revenue remains constant. According to our simulations, this corresponds to a specific excise at SAR 31. Even when we adopt a tax reform that keeps excise revenue constant, the change in tax structure is estimated to lead to a further 1% increase in average pack price, 1% reduction in sales, and 0.3% reduction in number of smokers with 0.1% reduction in prevalence. Setting a higher specific rate will lead to further reductions in sales and increases in tax revenue. If we set a higher specific excise (than the one that guarantees constant tax revenue), for example SAR 35, it is estimated that sales drop by 3% and total revenue increases by 10%.

In Table 3, we report only the (weighted by sales) change in price on average (increase of 1%). To gain some intuition, however, we need to look at what happens at the three price segments individually. Replacing the ad valorem tax with the specific tax, in a revenue neutral manner, has distinct effects on the three price segments. The average price of the premium segment decreases (− 3%) whilst the average price of mid-priced and economy segments increases (9 and 10%, respectively). As expected, the sales reduction comes from the mid-priced and economy brands. Due to the tax switch, the excise tax share of these segments increases and so does their contribution to the tax revenue. On average revenue is constant, as the increase in revenue from mid-priced and economy segments is offset by the revenue loss from the premium segment (due to the decrease in the excise tax share and their inelastic demand).

Overall, the three-year reform would lead to a higher than 50% increase in cigarette prices, 16% reduction in cigarette sales and almost 50% increase in total cigarette tax revenue. The final total tax share would be 81% and the excise share 76% of (all-taxes inclusive) final price.

Using the estimate that adult smokers were 2,676,978 in 2017 [20], and assuming an overall price elasticity of demand equal to − 0.3, we also estimated the number of deaths averted. Based on the standard estimate that the elasticity of smoking prevalence accounts for half of the total demand elasticity, that one in two of all regular smokers will die eventually, and that all quitters will survive [21], we estimated that 88,340 deaths related to cigarette smoking would eventually be averted due to the first year of the tax reform. That is, assuming a prevalence elasticity of − 0.15, a tax increase that would lead to a 44% increase in price, would lead eventually to a 6.6% reduction in cigarette-related deaths. Assuming a higher demand elasticity, of course, would lead to more deaths averted. For example, at a total demand elasticity equal to − 0.4, cigarette-related deaths would fall by 8.8% (117,787 deaths would eventually be averted).

### Sensitivity analysis

Our elasticity assumptions are rather conservative. Increasing cigarette demand elasticity per price segment, the estimated reduction in smoking prevalence is higher. Assuming, for example, a demand elasticity of − 0.3, − 0.4 and − 0.5 for premium, medium priced and economy brands respectively (scenario 1), smoking prevalence would fall by 1.4% (− 2.3% over the period of 3 years). Assuming, a demand elasticity of − 0.4, − 0.5 and − 0.6 for premium, medium priced and economy brands respectively (scenario 2), smoking prevalence would fall by 1.8% (− 2.5% over the period of 3 years).

Obviously, depending on the elasticity assumptions, there is a trade-off between a higher decrease in sales and hence the number of smokers and prevalence rate, and a lower increase in tax revenue. In scenario 1 and over the 3-year period, sales would fall by 21%, and excise tax revenue and total tax revenue would increase by 84 and 40%, respectively. In scenario 2 and over the 3-year period, sales would fall by 28%, and excise tax revenue and total tax revenue would increase by 75 and 32%, respectively.

Finally, assuming no trading down, we overestimate the reduction in sales and hence underestimate the increase in tax revenue. When, we allow for some trading down, that is, consumers turning to cheaper brands as prices go up, our results do not change significantly. In the absence of solid data, it is safer not to make any arbitrary assumptions on trading down or up.

## Discussion

The recent introduction of excise taxes by five of the six GCC countries, after five decades of sole reliance on custom duties, was a significant and major reform of the tobacco taxation policy. However, as a fulfilment of the GCC countries obligation under Article 6 of the WHO Framework Convention on Tobacco Control (FCTC) [22], tobacco taxation has to be aligned with the WHO recommendation that tobacco excise taxes account for at least 70% of the retail prices [3]. Simulations for Saudi Arabia show that a reform to this direction will lead to a significant increase in tax revenues and a reduction in cigarette use. The addition of VAT will contribute further to higher retail prices for cigarette products and higher tax revenues.

Our study examined a three-step reform, based on the WHO recommendations and best practices worldwide. Each step of the tax reform, and its results, indicate the significance of the implementation of these best practices. First, increasing the excise tax rate such that total tax share is at least 75% of the final consumer price would lead to substantial reduction in cigarette consumption. Second, replacing import duties with a higher excise compensates for revenue lost due to the global trend to reduce trade barriers. Introducing a MET guarantees a significant increase in price, especially in the lowest price segment, and hence in health benefits and public revenue. Finally, a shift towards specific taxation, even in a way that keeps revenue constant, leads to a further reduction in consumption.

Our simulations confirm the expected benefits from best practices. The tax reform, if implemented, is expected to lead to a reduction between 5 and 7.3% in number of smokers, and between 1 to 1.5% in smoking prevalence, over a 3-years period. Government revenues are expected to grow by 44 to 48%. The reform will result in a more robust GCC tax system and in line with WHO and FCTC recommendations [3, 22].

The tax base must be defined as clearly and as widely as possible for the tax to be more effective in reducing tobacco use and raising revenues. Packs of tobacco products can have a “maximum retail price”, stated on an affixed tax stamp, which also indicates excise has been paid in the particular country. This will facilitate identifying products illegally brought into the GCC countries. Saudi Arabia and UAE have started to implement such a tracking and tracing system in early 2019 and others are likely to follow similar procedures [23, 24].

Weak tax administration may lead to inefficiencies in tax collection and compliance when that tax is ad valorem, increasing the risk of tax avoidance and tax evasion [2]. This potential problem is one of the reasons, but not the main one, we recommend to gradually switch to specific excises and introduce a minimum excise floor [3, 14]. The European Union experience confirms that, even though price differentials still exist among member states, setting a minimum on the share of taxes in final price and a minimum excise tax, a certain level of approximation has been reached contributing to a declining trend in tobacco consumption and a stable trend in tax revenues [25].

A uniform specific tax is simple and raises price relatively more than an equivalent amount of ad valorem tax. Set at a high rate, specific taxes tend to reduce price dispersion and thus downward trading by the most vulnerable in society. In addition, specific taxes subdue manufacturers’ incentive to market low-priced products. It is important specific excises, including MET, be adjusted for inflation and income growth regularly, to ensure cigarettes do not become more affordable as income and inflation rise.

Governments should abolish duty free sales of tobacco products and cigarettes sold in packs of 10 or individual sticks, and small packets of other tobacco products such as waterpipe tobacco, as they accommodate affordability. Abolishing them will help preventing the youth and children from starting smoking. Manufacturers may be granted a short grace period to sell existing stock.

The size of the tax reform effects may vary across the GCC countries but their direction and implications will be the same, since they share similar market characteristics and harmonized import duties and taxes. Cigarettes is the most used tobacco product in all countries, with premium brands dominating the market and Marlboro being the most popular brand [10]. Although our analysis is based on cigarettes, we believe that the qualitative results can be generalized to all tobacco products in the GCC bloc. Given our proposals to increase taxes on all tobacco products, GCC governments should be vigilant of the repeated tobacco industry’s lobbying tactics of using the issue of smuggling in hindering implementation of tax reforms [26]. A recent publication on the interferences of the tobacco industry showed how industry representatives lobbied individual countries in the GCC to veto tax increments and defeat consensus on agreed resolutions of the Health Ministers’ Council [27].

### Limitations of the study

Our study has a few limitations. First, with the exception of Saudi Arabia, GCC countries did not have data on consumption on tobacco products by brands and types. Moreover, any available data related only to cigarettes and not the full range of tobacco products. However, we believe that our analysis applies equally to other tobacco products. Second, data on one country, Saudi Arabia, are used to generalize results of tax reforms to the other GCC states, had they applied the same tax structure and rates. Although, the quantitatively results may differ, their qualitative nature most certainly will hold.

## Conclusions

Our stepwise tax reform, involving changes in both tax rates and tax structure as well as phasing out of import duties, is expected to lead to significant improvements in both public health and tobacco tax revenues. The results provide useful insights that are of relevance to the whole GGC region. The effectiveness of the reforms, however, requires a strong tax and customs administration, including the establishment of a good database to monitor and advance public health.

Tax reforms must be supported by strengthened tax and customs administration, to ensure efficient and effective tax introduction and implementation. For a successful tax reform, data collection and sharing are paramount. Data collection is very important for understanding and addressing tobacco control. The GCC countries face great challenges in data collection and, hence, data analysis. Building a good administrative database, which can be regularly updated, will enable researchers to estimate accurate tobacco market features for each individual country and assess the impact of proposed tobacco policy reforms on both public health and public revenues. Policy makers can, then, make informed policy decisions. Political commitment facilitates coordination of all relevant agencies and contains resistance of vested interests. Hence, it accelerates reforms. Moreover, promoting national ownership of reform in collaboration with the WHO enables effective communication with shareholders and helps overcome their resistance, making clear what the potential benefits from the reforms, as well as the costs of maintaining the status quo, are.

## Data Availability

The main data (price and sales per cigarette brand), used for the baseline year of our simulations and supporting the findings of this study, are available from Euromonitor, under license, and hence are not publicly available. All tax data relevant to the study are included in the article and are published by the WHO. Further data would be available upon reasonable request from the Gulf Health Council.

## Abbreviations

CIF: Cost, Insurance and Freight
FTZ: Free Trade Zone
GCC: Gulf Cooperation Council
GDP: Gross Domestic Product
MET: Minimum Excise Tax
SA: Saudi Arabia
TIRSP: Tax-Inclusive Retail Sales Price
UAE: United Arab Emirates
VAT: Value Added Tax
WAP: Weighted Average Price
WHO: World Health Organization
